# Development of three-step holistic care pathways to detect and manage comorbidities in patients with atrial fibrillation: the Horizon 2020 EHRA-PATHS consortium

**DOI:** 10.1093/ehjopen/oeaf120

**Published:** 2025-10-27

**Authors:** Rana Önder, Lien Desteghe, Johan Vijgen, Rónán Collins, Rafal Dabrowski, Michal Miroslaw Farkowski, Marcio Jansen de Oliveira Figueiredo, Maartje J M Hereijgers, Daniel Hofer, Chu-Pak Lau, Geraldine Lee, Dominik Linz, Michelle Lobeek, Teresa Lopez, Christine McAuliffe, Jose Luis Merino, Tatjana Potpara, Prashanthan Sanders, Alireza Sepehri Shamloo, Maciej Sterliński, Emma Svennberg, Colinda van Deutekom, Isabelle Van Gelder, Michiel Rienstra, Hein Heidbuchel

**Affiliations:** Faculty of Medicine and Life Sciences, Hasselt University, Martelarenlaan 42, Hasselt 3500, Belgium; Heart Centre Hasselt, Jessa Hospital, Stadsomvaart 11, Hasselt 3500, Belgium; Faculty of Medicine and Life Sciences, Hasselt University, Martelarenlaan 42, Hasselt 3500, Belgium; Heart Centre Hasselt, Jessa Hospital, Stadsomvaart 11, Hasselt 3500, Belgium; Department of Cardiology, Antwerp University Hospital, Drie Eikenstraat 655, Edegem 2650, Belgium; Centre for Research and Innovation in Care (CRIC), Department of Nursing and Midwifery Sciences, University of Antwerp, Prinsstraat 13, Antwerp 2000, Belgium; Research Group Cardiovascular Diseases, GENCOR, University of Antwerp, Prinsstraat 13, Antwerp 2000, Belgium; Faculty of Medicine and Life Sciences, Hasselt University, Martelarenlaan 42, Hasselt 3500, Belgium; Heart Centre Hasselt, Jessa Hospital, Stadsomvaart 11, Hasselt 3500, Belgium; Department of Gerontology Trinity College and Tallaght University Hospital, Tallaght, Dublin D24 NR0A, Ireland; Department of Coronary Artery Disease and Cardiac Rehabilitation, National Institute of Cardiology, Alpejska 42, Warsaw 04-628, Poland; Department of Cardiology, Ministry of Interior and Administration National Medical Institute, Woloska 137, Warsaw 02-507, Poland; Department of Cardiology, University of Campinas (UNICAMP) Medical School, Campinas, R. Tessalia Vieira de Camargo 126, Sao Paulo 13083-887, Brazil; Department of Cardiology and Respiratory Medicine, Maastricht University Medical Centre +, P. Debyelaan 25, Maastricht 6229 HX, the Netherlands; Department of Cardiology, University Hospital Zurich, Rämistrasse 100, Zurich 8091, Switzerland; The Department of Medicine, The University of Hong Kong, Pok Fu Lam, Hong Kong SAR, China; School of Nursing & Midwifery, Brookfield Health Sciences Complex, University College Cork, Cork T12 AK54, Ireland; Department of Cardiology, Cardiovascular Research Institute Maastricht (CARIM), Maastricht University Medical Center, Universiteitssingel 50, Maastricht 6629 ER, The Netherlands; Department of Biomedical Sciences, Faculty of Health and Medical Sciences, University of Copenhagen, Blegdamsvej 3B, Copenhagen 2200, Denmark; Department of Cardiology, University of Groningen, University Medical Center Groningen, Groningen, The Netherlands; Department of Cardiology, Cardio-Oncology Unit, La Paz University Hospital, IdiPAZ Research Institute, Paseo de la Castellena 261, Madrid 28046, Spain; Cardio-Oncology Unit, Cardiology Department, Quironsalud Madrid, Diego de Velazques, Madrid 28223, Spain; Pharmacy Department, Tallaght University Hospital, Tallaght, Dublin D24 NR0A, Ireland; School of Pharmacy and Biomolecular Sciences, RCSI University of Medicine and Health Sciences, St Stephen's Green 111, Dublin D02 VN51, Ireland; Department of Cardiology, La Paz University Hospital, Paseo de la Castellena 261, Madrid 28046, Spain; Faculty of Medicine, Belgrade University, Dr. Subotica-starijeg 8, Belgrade 11000, Serbia; Cardiology Clinic, University Clinical Centre of Serbia, Pasterova 2, Belgrade 11000, Serbia; Centre for Heart Rhythm Disorders, University of Adelaide and Royal Adelaide Hospital, Adelaide, Australia; Department of Cardiology, Deutsches Herzzentrum der Charité-Medical Heart Center of Charité, German Heart Institute Berlin, Augustenburger Platz 1, Berlin 13353, Germany; 1st Arrhythmia Department, National Institute of Cardiology, Alpejska 42, Warszawa 04-628, Poland; Karolinska Institutet, Department of Medicine (MedH), Karolinska University Hospital Huddinge, Stockholm, Sweden; Department of Cardiology, University of Groningen, University Medical Center Groningen, Groningen, The Netherlands; Department of Cardiology, University of Groningen, University Medical Center Groningen, Groningen, The Netherlands; Department of Cardiology, University of Groningen, University Medical Center Groningen, Groningen, The Netherlands; Faculty of Medicine and Life Sciences, Hasselt University, Martelarenlaan 42, Hasselt 3500, Belgium; Department of Cardiology, Antwerp University Hospital, Drie Eikenstraat 655, Edegem 2650, Belgium; Research Group Cardiovascular Diseases, GENCOR, University of Antwerp, Prinsstraat 13, Antwerp 2000, Belgium

**Keywords:** Atrial fibrillation, Multimorbidity, Care pathways

## Abstract

**Aims:**

Older patients with AF (≥65 years) have on average four additional comorbidities. Comorbidity management requires a systematic approach for identification, and interdisciplinary care, often lacking in clinical practice. The EHRA-PATHS project’s overall aim is to create an approach to systematically address multimorbidity in older patients with AF.

**Methods and results:**

This project involves a consortium of 14 partners from 11 European countries. The comorbidity care pathways were developed using a stepwise approach. (i) A literature study. (ii) Online meetings/discussions to create structured care pathways. (iii) A two-round Delphi study for consensus on the final pathways (agreement ≥80%) and to rank the comorbidities for priority. (iv) Selection of comorbidities for evaluation in the planned randomized controlled trial (RCT). Development of care pathways for 23 comorbidities or special clinical settings was obtained and agreed upon. The Delphi surveys were sent to 37 consortium experts. After round 1 (28 responses), 13 pathways reached an agreement ≥80%. Twelve adjusted pathways were presented in round 2 (27 responses), of which 8 received an agreement ≥80%. The last four pathways were finalized after expert consensus. Hypertension, heart failure, and overweight were ranked as the most important comorbidities.

**Conclusion:**

A structured process of expert meetings and two Delphi rounds led to the development and ranking of 23 concise care pathways to identify and manage comorbidities in patients with AF. All pathways will be combined into a software tool, providing clinicians with a systematic approach to comorbidity management, which will be tested in the RCT of EHRA-PATHS.

## Graphical Abstract

**Graphical Abstract:**
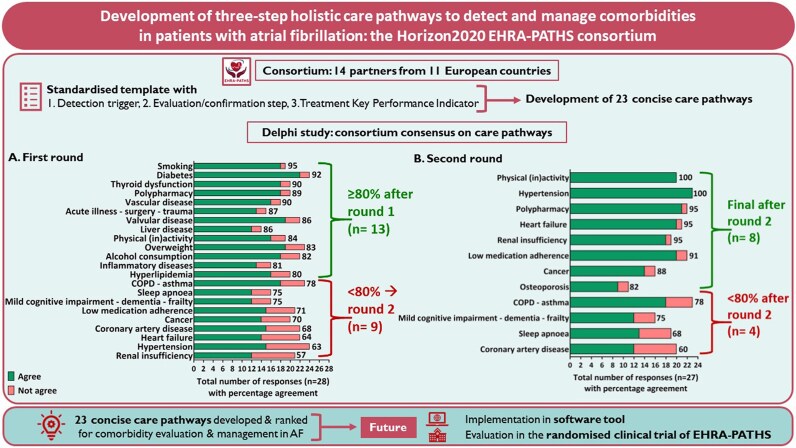

**Linked publication: Europace 2025; https://doi.org/10.1093/europace/euaf111.**

**Editorial for this article: Eur Heart J Open 2025; https://doi.org/10.1093/ehjopen/oeaf132.**

What’s new?

- Development of concise care pathways for 23 potential comorbidities in patients with atrial fibrillation (AF) with a uniform three-step structure:
  - Detection trigger
  - Confirmation testing
  - Managing the comorbidity within 6 months using key performance indicators
- Consortium consensus on the developed care pathways after a two-round Delphi study with an agreement target of ≥80%.
- Hypertension, heart failure, and overweight were ranked as the most important comorbidities to be considered in the management of older patients with AF.

## Introduction

Patients with atrial fibrillation (AF) are commonly diagnosed with several conditions and therefore categorized as having multimorbidity, which is mostly defined as the co-occurrence of two or more chronic conditions.^1,2^ Prior data have shown that 93.5% of patients with AF have at least one additional comorbidity and that older patients with AF (≥65 years) have an average of four comorbidities on top of AF.^1,3,4^ Hypertension, overweight, physical inactivity, excessive alcohol consumption, thyroid dysfunction, chronic kidney disease, chronic obstructive pulmonary disease, and sleep apnoea are some of the common comorbidities associated with AF.^1,5–11^ Comorbidities do not only contribute to the arrhythmia itself but also affect the outcomes of AF (i.e. impact on death, stroke, hospitalization, major bleeding, etc.) which was highlighted in a recently published review and previous studies.^12–14^ In addition, the treatment of comorbidities is also part of the new European Society of Cardiology (ESC) 2024 guidelines for AF management.^15,16^

Nonetheless, detecting and addressing multimorbidity systematically and effectively remains a challenge in daily clinical care. A pan-European survey from the EHRA-PATHS consortium investigated current practices among European Heart Rhythm Association (EHRA) members for managing multimorbid AF.^17^ A common theme is the perceived barriers for referring to specialist services for comorbidities due to the lack of an integrated care model (51%), organizational or institutional issues (43%), and issues with patient adherence (37%). The results confirm the need for developing tools for a systematic, more integrated management for multimorbid AF,^17^ ideally uniform among the European geography and beyond.

The EHRA-PATHS project was submitted in 2019 and awarded by the European Union’s Horizon2020 research and innovation programme (grant no 945260) in 2020.^18^ Its overall aim is to develop, implement, and evaluate systematic care pathways to tackle multimorbidity in older patients with AF. An important work package was to develop consensus on concise care pathways (i.e. practical and easy to implement) in order to systematically detect and address comorbidities or special clinical settings (all referred to below as ‘comorbidities’). This article outlines the development of these care pathways, which were later implemented in a software tool which is used in a randomized clinical trial (RCT).

## Methods

**Figure 1:**
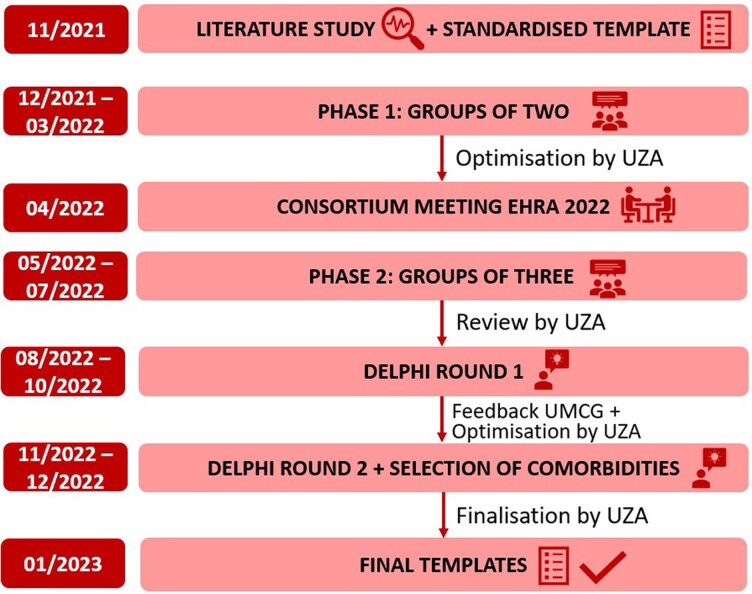
Phases towards the development of the care pathways. EHRA, European Heart Rhythm Association; UMCG, University Medical Centre Groningen; UZA, University Hospital Antwerp.

The EHRA-PATHS consortium comprises 14 partners (of which 12 clinical entities) from 11 European countries (see Supplementary material online, *Table S1*). The consortium is led by EHRA under the ESC umbrella. Each partner includes the expertise of at least one other sub-specialty besides cardiology (e.g. pneumology, endocrinology, gerontology, neurology, nephrology, oncology, primary care), allowing for truly multidisciplinary input during the care pathways development. The structure of the EHRA-PATHS project includes a plan divided into seven work packages over a 5 year timeline. The development of the care pathways was performed in work package 3 and included various steps between November 2021 and January 2023 (*Figure 1*). It was coordinated by the University Hospital Antwerp (UZA, Belgium).

### Literature study

An extensive literature study was performed to identify all possible relevant AF-related comorbidities, risk factors, or clinical settings relevant to AF management. The literature search was based on the ESC guidelines for AF management from 2020 and other references.^4,13,19–22^ Reasons for including a comorbidity were its recognition as a risk factor for AF,^19^ its potential impact on outcome of patients with AF, and its prevalence in patients with AF. Comorbidities were also proposed based on the first insights from the EurObservational Research Programme (EORP).^4,22^ The selection was discussed within the consortium at the outset of the project.

### Standardized template with a 6 month time horizon

A standardized template was created to set up a general structure for each comorbidity care pathway. The approach was that three steps needed to be completed within a 6 month time frame from the first consultation with a patient newly diagnosed with AF: (i) ‘detection trigger’: i.e. which minimal information is required to signal the potential presence of comorbidity?; (ii) ‘evaluation/confirmation’: i.e. which additional information is required to more formally confirm the presence of the comorbidity and/or its severity?; (iii) ‘treatment Key Performance Indicator (KPI)’, i.e. how can be confirmed that a treatment pathway has been initiated (e.g. by appropriate referral, start of treatment) or treatment goal attained? For most comorbidities, the achievement of a final treatment goal was not reasonable given the 6 month time horizon. In the planned RCT, detection and implementation within this 6 month interval will be evaluated.

The templates were uploaded on an online collaborative platform on the private and protected EHRA-PATHS website, allowing the consortium partners to work together on a first draft of the intentioned care pathways.

### First drafts of care pathways through online discussion meetings

In total, 23 comorbidity care pathways have been developed, 22 of which were based on the initial literature study and one added later (see below). The consortium partners worked in subgroups (defined by the coordinator UZA based on the partners’ multidisciplinary expertise) to create the first proposals for the care pathways in two phases.

**In Phase 1**, partners were asked to provide suggestions for the three template steps for a number of assigned comorbidities. Each comorbidity was assigned to two partners, i.e. each partner had to discuss four to six comorbidities (but were free to contribute to any other comorbidity if they wished to do so). Partners were asked to provide together a first consensus draft.

After this phase, all the care pathway templates were evaluated by the UZA research team on (a) whether they adhered to the three-step concept and (b) were concise and specific enough to be workable in clinical practice. Criteria were as follows: (i) as simple as possible, (ii) implementable in a software tool with embedded logic, (iii) using specific input (e.g. exact laboratory tests or echocardiogram parameters), (iv) clarity on whether evaluation elements are needed in all patients or in specific subsets, and (v) clarity on ‘and’ or ‘or’ options if multiple items are needed to be checked. In an interim phase, some care pathways were presented during an interactive session at a **consortium meeting** in 2022, to illustrate and converge on a common conceptual approach. In follow-up to this meeting, partners were requested to optimize the templates.

At the consortium meeting, it was also decided to formally re-assign the comorbidities to other partners for them to discuss and optimize in a **second phase**. The re-assignment was done in groups of three, and the partners were asked to discuss five or six comorbidity templates (i.e. four groups with three partners). The results were again reviewed and optimized by UZA along the five criteria mentioned above to ensure practical use in a clinical setting, with clear phrasing and a simple layout for later software implementation.

### Two-round Delphi study within the consortium

A Delphi study was set up in order to obtain ≥80% consensus for each care pathway by the consortium members and representatives of the Patient Advisory Board. Two online rounds were conducted with the use of Qualtrics (Qualtrics XM, USA).

In the **first round**, all consortium partners were asked specific questions on the remaining issues of the different comorbidity care pathways and/or whether they agreed with the current comorbidity care pathway (besides the questions raised). Each participant was allowed to withhold from voting for individual pathways if they deemed themselves not qualified to give input. If participants did not agree on a proposed care pathway, they were asked for suggestions on how they wanted to optimise the care pathway. An agreement level of ≥80% was required to consider a comorbidity care pathway as approved by the consortium. Otherwise, the comorbidity needed to go to the second round. Before that, the UZA team adapted those care pathways based on the provided input from round one, often after bilateral discussions with specific experts from the consortium.

Between the first and second Delphi round, a **selection process occurred of the comorbidities that would be the focus of the planned RCT**. Given the number of comorbidities, it was envisioned that it would not be feasible to test the effectiveness of all newly developed care pathways in the RCT. Therefore, the coordinator of the RCT, University Medical Centre Groningen, made a proposal for a group of comorbidity pathways to be evaluated as the primary endpoint in the RCT. The proposal was made based on the recognition of the clinical importance of different comorbidities in the 2020 ESC guidelines on the management of AF, on the first insights from the EurObservational Research Programme (EORP),^19,23^ and on the feasibility for practical and unambiguous evaluation of the care pathways as part of the primary endpoint of the RCT. The primary endpoint is the proportion of risk factors and comorbidities that are identified and for which treatment is initiated during base mapping (Part 1) and at the end of the RCT (Part 2).

The **second Delphi round** was performed with three aims. First, the partners were asked again for consensus on the outstanding pathways (or further suggestions for adaptation if they did not agree with the proposal). They were also asked to rank the comorbidities in order of importance, based on their perceived impact on the outcome of patients with AF and their prevalence. Finally, they were asked if they approved with the proposed selection of the comorbidities for the RCT.

After this round, the UZA team in concert with experts decided on a final formulation of the four care pathways, which did not yet reach the 80% agreement criterion, as discussed below.

## Results

### Development of the concise care pathways with three steps

**Figure 2:**
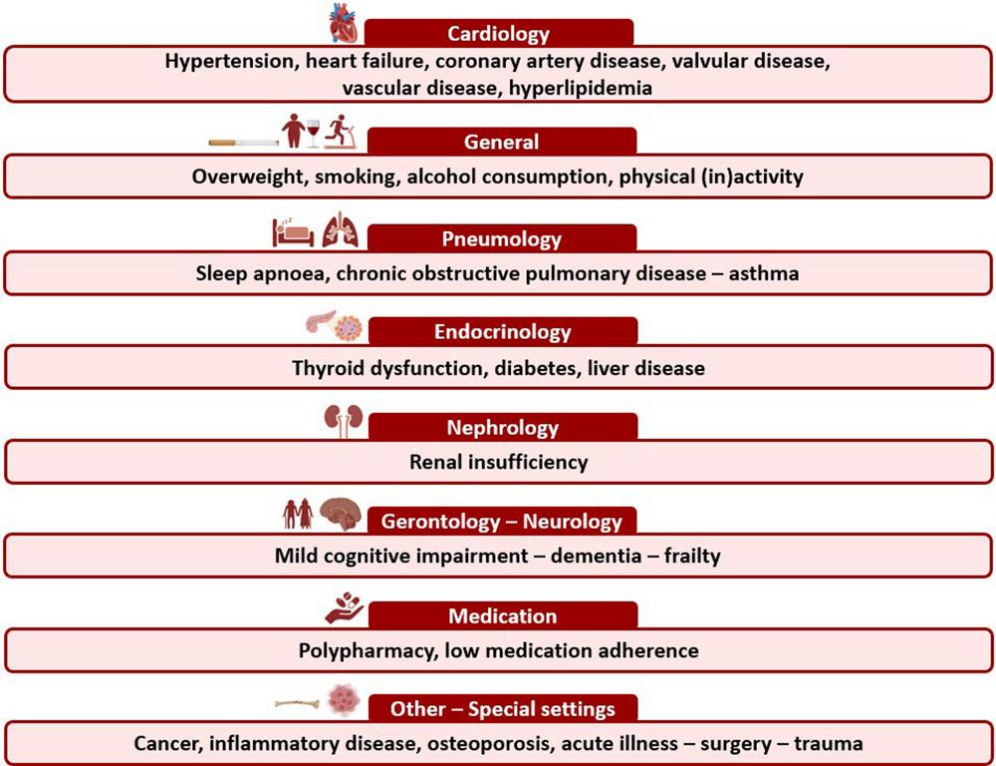
The 23 final EHRA-PATHS comorbidities grouped by category.

Twenty-two comorbidities were defined in the initial literature search, and during the first Delphi round, one comorbidity was added (osteoporosis). Thus, care pathways were ultimately developed for 23 AF-related comorbidities deemed relevant in patients with AF. The comorbidities can be divided into eight different categories (*Figure 2*): cardiovascular comorbidities, general risk factors, pneumology, endocrinology, nephrology, gerontology–neurology, medication, and other–special settings.

**Figure 3:**
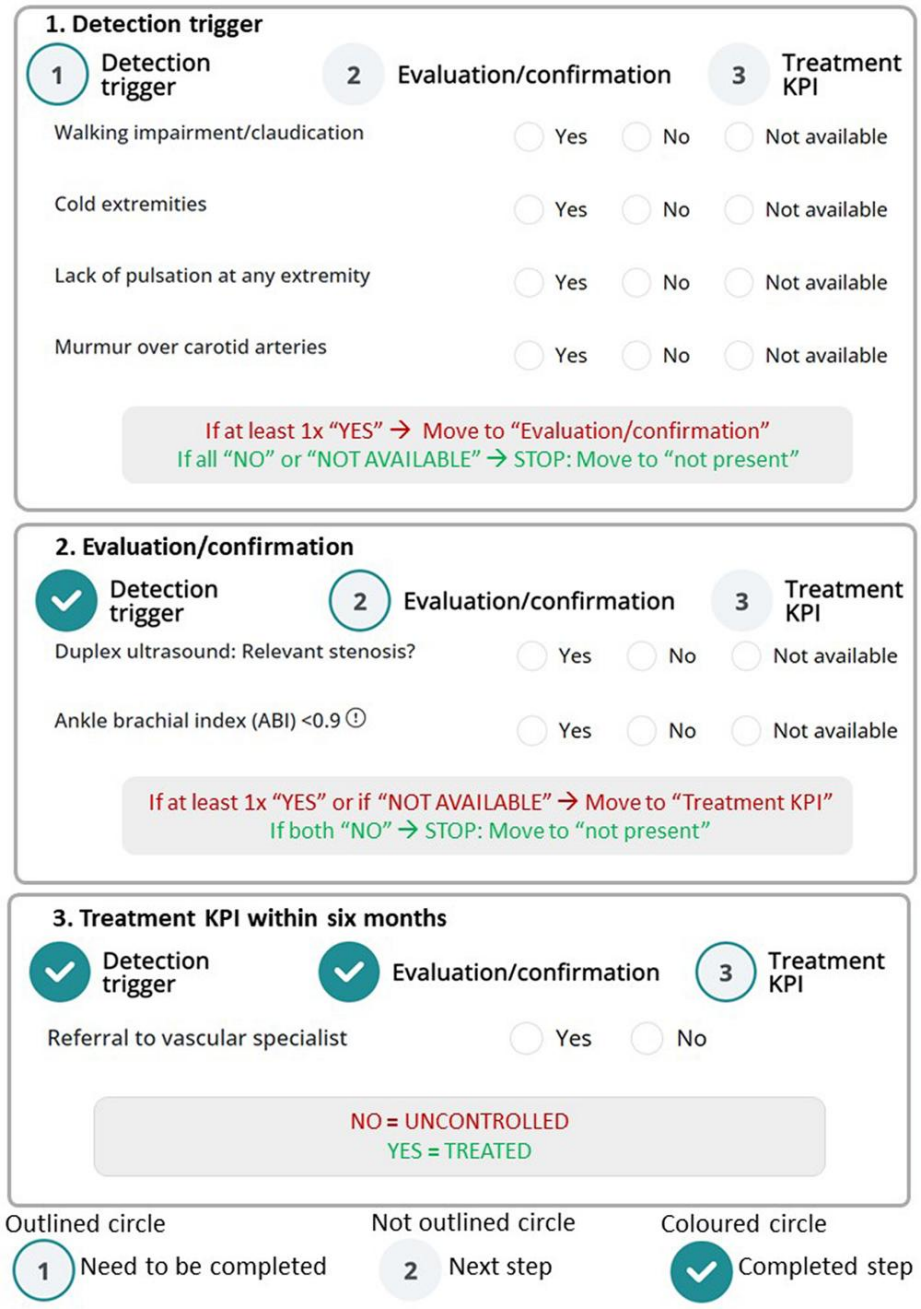
Example of a concise care pathway, for the comorbidity ‘vascular disease’. Screenshot of the pathway as implemented in the software tool. The windows for ‘detection trigger’, ‘evaluation/confirmation’, and ‘treatment KPI’ are shown. The later windows will only appear when in a detection trigger suggests that there might be vascular disease, which needs confirmation. If confirmed ‘present’, an adequate referral should be done within at least 6 months. The light grey box includes instructions to further clarify how the content and logic of the care pathways should be implemented for the software developers.

Given the ongoing clinical trial, we cannot disclose the final care pathways of the RCT-tested comorbidity pathways, but *Figure 3* shows an example for ‘vascular disease’, which was not selected as part of the primary endpoint (but will be available for participating centres for use if they wish to do so). This is a screenshot of the pathway as implemented in the web-based software tool, with the three phases of ‘detection’, ‘confirmation’, and ‘treatment KPI’, which should be evaluated within the first 6 months from consultation with a patient newly diagnosed with AF. The software only shows the relevant following phases when needed. That is, if the presence of a comorbidity can be ruled out in step 1 or 2, a treatment pathway is not needed, and the comorbidity will be listed as ‘not present’. In this way, the software allows a rapid overview of comorbidities that need further evaluation, excluded comorbidities, and those in which treatment needs to be initiated or evaluated. The GDPR- and MDR-proof software can be accessed on a server via a web browser (which may be embedded in existing medical applications in a later phase), after authentication for different authorization levels. Users can download structured information on their patients for integration in the hospital EMR, if needed. The team of the UZA compiled a manual with instructions for using the software. Software testing and feedback on the manual have been performed by all consortium experts. A separate article, published concomitantly, describes in more detail software development, features, and testing (doi number: 10.1093/europace/euaf111).^24^

### Delphi study: consortium consensus on the developed care pathways

**Figure 4:**
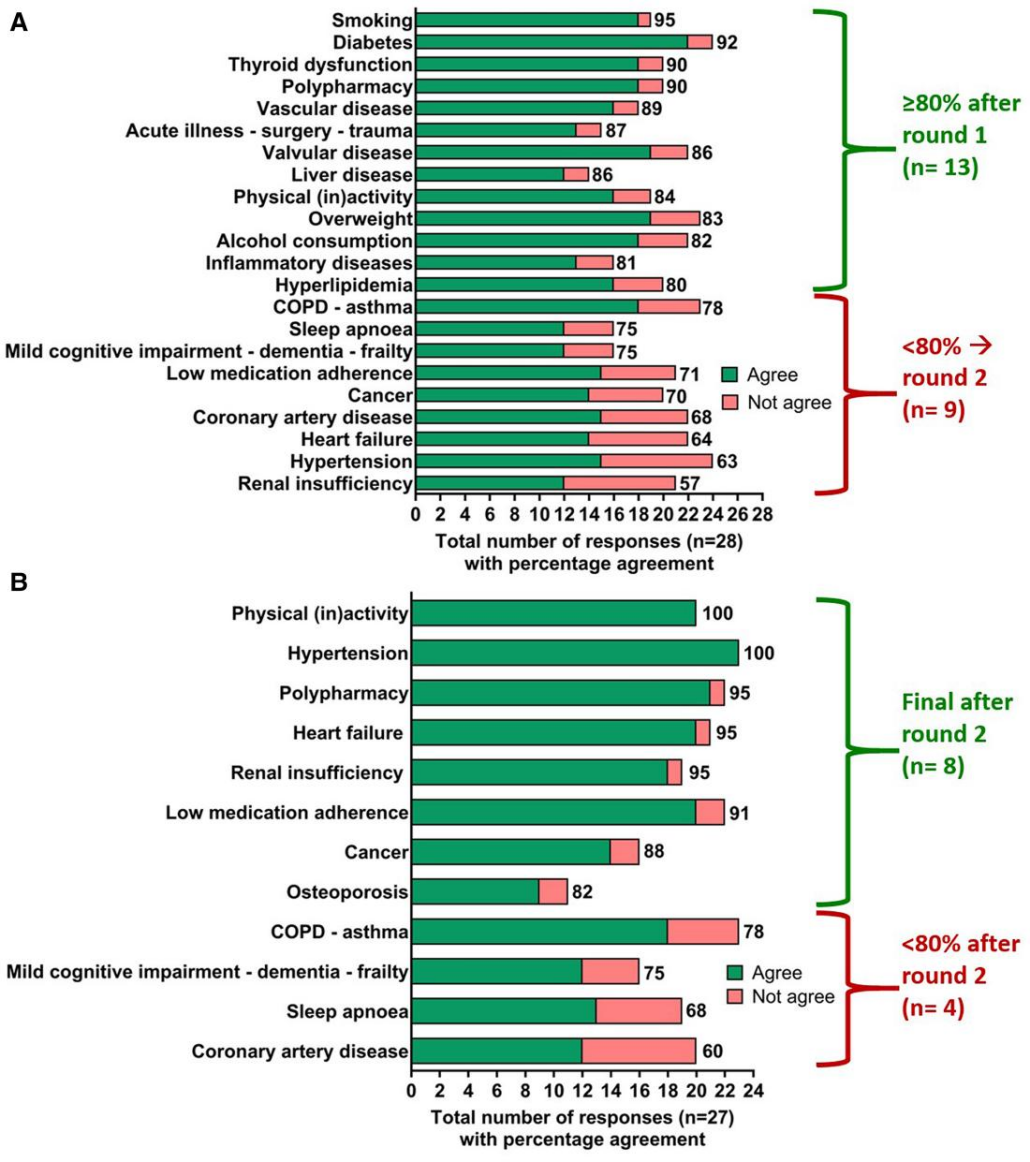
Two-round Delphi study to find consensus on the care pathways. (*A*) Delphi round 1: votes by 14 to 24 of the 28 respondents, with percentage agreement for each comorbidity care pathway. (*B*) Delphi round 2: votes by 11 to 23 of the 27 respondents, with percentage agreement for each comorbidity care pathway. COPD, Chronic obstructive pulmonary disease.

Round 1 from the Delphi survey was sent to 37 experts from the consortium (cardiology and other subspecialties). Twenty-eight (75.7%) replied, with each comorbidity care pathway completed by at least 14 experts (50.0%). ‘Overweight’ was the most frequently completed comorbidity (25 experts, 89.3%). After round 1, 13 comorbidity care pathways (59.1%) had an agreement of ≥80% and could be accepted with only minor changes (*Figure 4A*), while 9 comorbidities (40.9%) had an agreement of <80%. However, since some agreed comorbidity care pathways could be further optimized with the feedback from the experts [e.g. physical (in)activity and polypharmacy], and one extra comorbidity was defined (osteoporosis), a total of 12 comorbidities were presented during Delphi round 2. Twenty-seven of 37 invited experts replied (73.0%), with each comorbidity reviewed by at least 11 experts (40.7%). Hypertension and chronic obstructive pulmonary disease–asthma were the most frequently completed comorbidities during this round (23 experts; 85.2%). Eight of the 12 comorbidity care pathways (66.7%) found an agreement of ≥80%, while the 4 other comorbidity care pathways [chronic obstructive pulmonary disease (COPD)–asthma, mild cognitive impairment–dementia–frailty, sleep apnoea, and coronary artery disease] had agreements between 60 and 78% (*Figure 4B*). All care pathways were further optimized with the feedback received, while the final four comorbidity care pathways were finalized after further discussion with specific experts reaching a consensus during online discussions that were set up by the coordinator UZA.

The 27 experts of round 2 also ranked the comorbidities for clinical importance (i.e. taking into account their prevalence and impact on AF progression, symptoms, or prognosis) in patients with AF. Supplementary material online, *Table S2* shows that hypertension, heart failure, and overweight were ranked as the most important ones in the group of the RCT-selected comorbidities. Sleep apnoea, low medication adherence, and mild cognitive impairment–dementia–frailty were the top non-RCT selected comorbidities.

## Discussion

Work package 3 of the EHRA-PATHS project developed 23 structured care pathways to evaluate and manage comorbidities in patients with AF. The envisioned aim was to develop a tool allowing to systematically evaluate the presence or absence of a comorbidity and to follow up on the effective implementation of its multidisciplinary management. We aimed for a time horizon of 6 months after the first consultation with a patient newly diagnosed with AF to complete this structured and systematic evaluation. A three-step approach was devised, with care pathways created and unanimously approved by the consortium for each of the comorbidities.

### Previous studies on structured comorbidity management in AF

In the last years we have seen an increased interest in ‘integrated care’ for AF as one of the major chronic conditions in modern society. According to the World Health Organization, integrated care is described as a set of ‘health services that are managed and delivered so that people receive a continuum of health promotion, disease prevention, diagnosis, treatment, disease-management, rehabilitation and palliative care services, coordinated across the different levels and sites of care within and beyond the health sector, and according to their needs throughout the life course’.^25^ It is recognized that addressing comorbidities is an essential component of integrated care.^2^ Prior studies on integrated care in AF have used many different approaches overall and concerning comorbidity management in particular. The outcomes of these trials and the effect on endpoints including mortality and hospitalizations have been mixed,^13,14,26–44^ which may be partly due to variable approaches towards a variable set of comorbidities, as is summarized in *Table 1*. The table shows that a maximum of 13 comorbidities were evaluated in these trials, while our consortium defined 23 relevant comorbidities for which specific care pathways were devised. Moreover, it is recognized that there is a lack of information on optimal AF management in specific populations, such as those with cancer, chronic kidney disease, cognitive impairment, and frailty.^45^ Limited efforts have been made for a truly systematic detection/evaluation/tracking of comorbidities in patients with AF although this is a crucial pillar within AF care according to AF Guidelines worldwide (e.g. the ‘C’ of ‘cardiovascular and comorbidity risk optimisation’ in the 2020 and 2024 ESC Guidelines).^15,19,46^ Moreover, the AF-EduCare trial demonstrated that intensive targeted patient education did not lead to a decrease in unplanned cardiovascular events in the AF patient population.^47^ On the other hand, like in a study conducted by Hendriks *et al.*, it showed that integrated specialized AF clinics reduce all-cause mortality.^48^ The recently ended ESC/EHRA STEEER-AF trial, deployed across six European countries and recruiting 1732 patients with AF (mean age of 69 years), has shown important gaps in guideline adherence in real-world practice.^44^ The study later studied the impact of a structured educational programme for healthcare professionals on guideline adherence and patient outcomes (results are pending). Therefore, more concrete and precise pathways are required. This was also mentioned in the foregoing European EHRA-PATHS survey on current management barriers and clinical priorities for managing multimorbid AF.^17,44^

**Table 1:** Comorbidities addressed in published integrated care AF studies

Article	Groups	Setting	Number of addressed comorbidities	Addressed comorbidities
1. Wijtvliet *et al.* (RACE 4 Trial)^37^	Nurse-led vs. usual care	Outpatient cardiology clinic	6 parameters	This paper focused on the following parameters and not specifically the comorbidities: Oral anticoagulation, echo, blood pressure, thyroid stimulating hormone, creatinine, and glucose
2. Tenbult *et al.*^29^	Cardiac rehabilitation intervention	Cardiac rehabilitation unit	1	Overweight/obesity
3. Kotalczyk *et al.* (ChiOTEAF Registry)^30^	ABC group vs. Non-ABC group	Consecutive patients presenting to cardiology, neurology, or surgical services	13	Diabetes, hypertension, heart failure, coronary artery disease, liver disease, lipid disorder, prior ischaemic stroke, chronic kidney disease, chronic obstructive pulmonary disease, sleep apnoea, dementia, prior major bleeding, and polypharmacy
4. Esteve-Pastor *et al.* (FANTASIIA Registry)^31^	ABC group vs. Non-ABC group	Consecutive outpatients	6	Hypertension, diabetes mellitus, heart failure, coronary artery disease, peripheral artery disease, and stroke/transient ischaemic attack
5. Rivera-Caravaca *et al.* (Murcia AF Project Phase II Cohort)^32^	ABC group vs. Non-ABC group	Outpatients	6	Hypertension, coronary artery disease, peripheral artery disease, heart failure, stroke/transient ischaemic attack, and diabetes mellitus
6. Patel *et al.* (ENGAGE AF-TIMI 48 trial)^33^	ABC group vs. Non-ABC group	No specific setting mentioned	8	Hypertension, coronary artery disease, peripheral artery disease, stroke/transient ischaemic attack, diabetes mellitus, heart failure with reduced ejection fraction, alcohol consumption, and smoking
7. Romiti *et al.* (GLORIA-AF Registry)^34^	ABC group vs. Non-ABC group	Consecutive patients in hospitals, anticoagulant clinics, specialists and general practice settings	9	Hypertension, diabetes, coronary artery disease, congestive heart failure, history of previous stroke/transient ischaemic attack, peripheral artery disease, dyslipidaemia, dyspeptic disease, and hyperthyroidism
8. Guo *et al.* (ChioTEAF Registry)^35^	ABC group vs. Non-ABC group	Consecutive patients presenting to cardiology, neurology, or surgical services	7	Hypertension, coronary artery disease, peripheral artery disease, previous ischaemic stroke, heart failure, diabetes mellitus, and lipid disorders
9. Proietti *et al.* (ESC-EHRA EORP-AF long-term general registry)^13^	ABC group vs. Non-ABC group	Cardiology services	6	Hypertension, coronary artery disease, peripheral artery disease, previous stroke/transient ischaemic attack, heart failure, and diabetes mellitus
10. Ding *et al.* (ESC-EHRA EORP-AF long-term general registry)^36^	ABC group vs. Non-ABC group	Cardiology services	6	Hypertension, coronary artery disease, peripheral artery disease, previous stroke/transient ischaemic attack, heart failure, and diabetes mellitus
11. Krittayaphong *et al.* (COOL-AF registry)^14^	ABC group vs. Non-ABC group	Cardiology services	5	Hypertension, coronary artery disease, ischaemic stroke, heart failure, and diabetes
12. Pathak *et al.* (ARREST-AF cohort study)^38^	RFM group vs. controls	Consecutive patients undergoing AF ablation	7	Blood pressure control, weight management, lipid management, glycaemic control, sleep-disordered breathing management, smoking, and alcohol
13. Pathak *et al.* (LEGACY study)^39^	Group 1:≥10% weight loss vs. group 2: 3% to 9% weight loss vs. group 3:<3% weight loss	Consecutive patients who were referred for management to cardiology Heart Rhythm Disorders Centre	7	Blood pressure control, weight management, lipid management, glycaemic control, sleep-disordered breathing management, smoking, and alcohol
14. Middeldorp *et al.* (REVERSE-AF study)^40^	Group 1:≥10% weight loss vs. group 2: 3% to 9% weight loss vs. group 3:<3% weight loss	Consecutive patients who were referred for management to cardiology Heart Rhythm Disorders Centre	7	Blood pressure control, weight management, lipid management, glycaemic control, sleep-disordered breathing management, smoking, and alcohol
15. Abed *et al.*^41^	Intervention (physician-led weight loss programme) vs. control (self-directed general lifestyle measures)	Patients from the Centre for Heart Rhythm Disorders	7	Weight management, hypertension, hyperlipidaemia, glucose intolerance, sleep apnoea, alcohol, and smoking
16. Voskoboinik *et al.*^42^	Abstinence from alcohol group vs. control group (continue their usual alcohol consumption)	Patients from six tertiary hospitals (no specific setting mentioned)	3	Alcohol, hypertension, and overweight
17. Lee *et al.*^43^	4 groups: (i) never smokers, (ii) ex-smokers, (iii) smoking cessation after AF diagnosis (‘quitters’), and (iv) current smokers	Patients from the Korean National Health Insurance Service database	1	Smoking
18. Sterlinski *et al.* (ESC and EHRA STEER-AF Trial)^44^	Intervention (structured educational programme for healthcare professionals treating patients with AF) vs. control (routine practice with no added education)	Hospital units treating patients with AF on a daily basis	6	Hypertension, heart failure, diabetes, coronary artery disease/prior myocardial infarction, history of stroke or transient ischaemic attack, diagnosis of COVID-19

ABC, Atrial Fibrillation Better Care.

Moreover, other similar H2020 trials are ongoing, encompassing attention for multimorbidity in AF. The AFFIRMO project (Atrial Fibrillation Integrated Approach in Frail, Multimorbid, and Polymedicated Older People) started in May 2021 and seeks to improve care for older patients with AF by focusing on the ABC pathways.^49^ The project develops a digital platform to assist HCPs in decision-making while also evaluating the relationship between AF and other comorbidities. No data have been published so far, however. In their RCT, the outcome of two groups of 1200 multimorbid patients with AF across 48 hospitals [one receiving a structured digital care pathway (iABC group) and the other standard care from doctors (Usual Care group)] will be compared. The H2020 ARISTOTELES trial (started in November 2023) aims to develop and validate an AI-based approach within integrated care systems to improve the management of AF and other comorbidities.^50^ This AI-based tool seeks to enable personalized risk assessment and targeted management strategies to reduce complications, hospitalization, and mortality. A cluster-RCT will be performed in patients with AF, who will be randomized to usual care or AI-supported care. No data have been published yet.

### The current EHRA-PATHS approach

Realizing a scope of 23 comorbidities, it was clear that to be implemented into clinical practice, the care pathways needed to be concise and easy to use. Therefore, considerations during the creation of the care pathways were that (i) detection triggers needed to be specific on everything (e.g. exact lab test values, echo parameters, questions), (ii) were clear on whether certain evaluations were needed in all patients or only in subsets, and (iii) were logical in the proceeding to the next step for confirmation and start of management/referral and (iv) the treatment KPI should be achievable within 6 months. Full achievement of treatment goals is not necessarily desired (e.g. reaching a target weight in case of obesity), but actions to get there needed to be implemented (e.g. referral to a structured weight loss programme or referral to another speciality). This approach can be extended to future annual reassessments. To ensure a comprehensive assessment in all new patients with AF, the care pathways were later implemented in a new software tool that can guide healthcare providers (i.e. physicians and AF nurse specialists) through the different care pathways and provide a systematic overview of all the comorbidities in a given patient with AF. The simple concept of the care pathways makes this software tool feasible for use in busy clinical settings. An ongoing study is evaluating the time needed to complete the care pathways. The software tool has an architecture by-design which allows it to be integrated with hospital electronic health records in the future.

### Limitations of the care pathway development process used

A limitation of this project with a tight deadline and restrained resources is the fact that no one outside the EHRA-PATHS consortium was able to provide feedback on the 23 care pathways, except via the Worldwide Scientific Advisory Board who provided input during consortium meetings. This limitation was essential to avoid bias for the healthcare providers who would later be involved in the RCT. Nevertheless, the required expert knowledge was guaranteed as all relevant subspecialties were represented in this consortium (e.g. pneumology, rehabilitation, gerontology–neurology, nephrology, primary care). These experts had ample discussions on the initial drafts of specific care pathways, while the coordinating centre UZA provided a unified template for guidance, critical intermediary review and feedback, and open discussion during plenary consortium meetings. Not all 37 experts reviewed all care pathways during the Delphi study, since they did not always consider themselves qualified for other comorbidities. Nevertheless, we feel that a sufficient number of votes were submitted for confident Delphi consensus assessment. On the other hand, not all comorbidities may have the same impact for an individual patient. Therefore, it remains clinically important to focus on the specific needs of each patient to improve their quality of life. Current guidelines also mention the importance of psychological disorders, such as depression or anxiety, which now is not included in our current list but could certainly be added in the future. The planned RCT will evaluate depression and anxiety using questionnaires. Furthermore, while the pathways are designed to be concise, their implementation may face challenges such as resource constraints, training needs, and variations in national systems. The software is being conceived so that care pathways can be adjusted to local and national realities. That implementation was limited in the RCT for obvious reasons, but it will be possible when the software is later made available to a much broader user base.

### Future perspectives

All care pathways have now been implemented into a novel software tool which will form the core for evaluation of the care pathways in the RCT of EHRA-PATHS, coordinated by the University Medical Centre Groningen (ClinicalTrials.gov Identifier: NCT05773768). The RCT will be performed in 11 European countries (i.e. Austria, the Netherlands, Belgium, Bulgaria, Croatia, France, Germany, Greece, Italy, Poland, and Portugal) with 5 to 6 participating centres each. None of these centres is part of the consortium. The RCT will provide a wealth of user feedback from use of the software in real-life, allowing for later improvements. The aim of this study is to evaluate whether the use of the EHRA-PATHS care pathway approach in daily practice will lead to better detection and more effective management implementation of risk factors and comorbidities, compared with current standard care. Experience from the ongoing clinical study using these care pathways in daily care throughout Europe will certainly provide valuable information for later adjustments.

## Conclusions

Via a coordinated process of template setting, expert discussions, plenary review, and two Delphi rounds, we developed 23 concise care pathways to systematically detect and manage comorbidities in patients with AF presenting in daily clinical practice. Each care pathway has a three-step approach, with a detection trigger, an evaluation/confirmation step, and a treatment KPI which should be achieved within a 6 month time frame. All care pathways have been implemented into a novel software tool, and their effectiveness will be evaluated in the RCT of EHRA-PATHS. We envision that our efforts result in more uniform comorbidity evaluation, helping healthcare providers not only in the RCT but later also during clinical work-up, leading to improved outcomes for patients with AF throughout Europe and beyond.

## Supplementary Material

oeaf120_Supplementary_Data

## Data Availability

No new data were generated or analysed in support of this research.
