# Supplementary material for: Development of three-step holistic care pathways to detect and manage comorbidities in patients with atrial fibrillation: the Horizon 2020 EHRA-PATHS consortium
Source: Eur Heart J Open. 2025 Oct 27;5(5):oeaf120. doi: 10.1093/ehjopen/oeaf120 (PMC12555002; doi:10.1093/ehjopen/oeaf120)
Supplement: oeaf120_Supplementary_Data [file oeaf120_supplementary_data.docx]

Supplementary tables

**Supplementary Table 1**

| Participant organization name | Country | Subspecialty |
| --- | --- | --- |
| European Society of Cardiology/ European Heart Rhythm Association *(Coordinator)* | France | NA |
| Antwerp University Hospital | Belgium | Pneumology |
| University of Hasselt | Belgium | Cardiac rehabilitation |
| University Medical Centre Groningen | Netherlands | Gender aspects, health economics |
| Academic Hospital Maastricht | Netherlands | Sleep disordered breathing |
| Leipzig Heart Institute GMBH | Germany | Public health systems |
| University of Belgrade (Faculty of Medicine) | Serbia | Endocrinology |
| University Hospital of Zürich | Switzerland | Primary care |
| Karolinska University Hospital | Sweden | Nephrology |
| University College Cork | Ireland | Patient-centred care |
| Tallaght University Hospital | Ireland | Gerontology–Neurology |
| National Institute of Cardiology of Stefan Cardinal Wyszynski | Poland | Polypharmacy–Therapy adherence |
| Servicio Madrileno De Salud | Spain | Oncology |
| Catalyze BV | Netherlands | NA |

**The 14 partners of the EHRA-PATHS consortium**

For each partner, the non-cardiology subspecialty involved in conjunction with the local cardiology department is listed. NA: Not applicable

**Supplementary Table 2**

**Ranking of the RCT-selected and non-RCT selected comorbidities during Delphi round 2**

| RCT-selected comorbidities | Non-RCT selected comorbidities |
| --- | --- |
| 1. Hypertension | 1. Sleep apnoea |
| 1. Heart failure | 1. Low medication adherence |
| 1. Overweight | 1. Mild cognitive impairment – dementia – frailty |
| 1. Alcohol consumption | 1. Thyroid dysfunction |
| 1. Valvular disease | 1. Polypharmacy |
| 1. Coronary artery disease | 1. Cancer |
| 1. Renal insufficiency | 1. Inflammatory disease |
| 1. COPD – asthma | 1. Vascular disease |
| 1. Diabetes | 1. Liver disease |
| 1. Physical (in)activity | 1. Osteoporosis |
| 1. Smoking | 1. Acute illness – surgery – trauma (special setting) |
| 1. Hyperlipidemia |  |

The comorbidity ‘Acute illness-surgery, trauma’, is depicted in grey colour since this comorbidity refers to special circumstances. It is not a standard comorbidity to evaluate when a new patient with AF presents; it will only need to be evaluated in special situations, like during an emergency admission or if the patient has been referred for surgery. COPD: Chronic obstructive pulmonary disease; RCT: Randomised Clinical Trial.
